# The Potential Contribution of ABO, Lewis and Secretor Histo-Blood Group Carbohydrates in Infection by *Toxoplasma gondii*

**DOI:** 10.3389/fcimb.2021.671958

**Published:** 2021-06-18

**Authors:** Luiz Carlos De Mattos, Ana Iara Costa Ferreira, Karina Younan de Oliveira, Fabiana Nakashima, Cinara Cássia Brandão

**Affiliations:** ^1^ Immunogenetics Laboratory, Molecular Biology Department, Faculty of Medicine – FAMERP, São José do Rio Preto, Brazil; ^2^ FAMERP Toxoplasma Research Group, Molecular Biology Department, Faculty of Medicine – FAMERP, São José do Rio Preto, Brazil

**Keywords:** histo-blood group systems, carbohydrates, secretor, lewis phenotype, secretor phenotype, ABO blood group, *Toxoplasma gondii*

## Abstract

The glycosyltransferases encoded by genes from the human ABO, Lewis, and Secretor histo-blood group systems synthesize part of the carbohydrate antigens in hematopoietic and non-hematopoietic tissues. The combined action of these glycosyltransferases strongly influences cell, tissue, mucosa, and exocrine secretion carbohydrate phenotypes, including those serving as habitat for mutualistic and pathogenic microorganisms. A set of reports investigated associations between *Toxoplasma gondii* infection and the ABO histo-blood group system, but the results are contradictory. As *T. gondii* uses the gastrointestinal tract as a route for infection, and in this organ, the expression of ABO, Lewis, and Secretor histo-blood group carbohydrates occurs, it is reasonable to suppose some biological relationship between them. This text reviewed association studies published in recent decades focusing on the potential contribution of the ABO, Lewis, and Secretor histo-blood group carbohydrates and infection by *T. gondii*.

## Introduction

*Toxoplasma gondii*, the etiologic agent of toxoplasmosis, was first identified in 1908 by Alphonso Splendore, in São Paulo, Brazil, in tissues of a rabbit, and by Nicolle and Manceaux in Tunisia, North Africa, in the tissues of a rodent *Ctenodoactylus gundi* (Ferguson, 2009; Innes, 2010). According to the current taxonomic classification, this parasite belongs to the Phylum Apicomplexa, which contains other pathogens such as *Plasmodium, Theileria, Eimeria, Neospora, Cryptosporidium*, and *Babesia* species (Arisue and Hashimoto, 2015).

*T. gondii* presents a heteroxenous life cycle, with felines being the definitive hosts. All warm-blooded animals, including humans, are intermediate hosts. The life cycle of *T. gondii* has three different developmental stages. Tachyzoites, the free form found in the peripheral blood during the acute phase of infection, can cross the placenta, reaching the fetus during pregnancy, allowing congenital toxoplasmosis. Bradyzoites, which form tissue cysts and remain in the latent stage, can infect humans by raw or undercooked meat consumption. Oocysts, shed in the feline’s feces, constitute the environmentally resistant stage of *T. gondii* and can infect humans by ingesting contaminated water and vegetables (Dubey, 2009).

*T. gondii* is a cosmopolitan obligatory intracellular parasite that infects all nucleated cells from warm-blooded animals (Furtado et al., 2011; Subauste et al., 2011). It presents tropism for some human organs and tissues such as the brain, choroid, retina, as well as placenta (Dadimoghaddam et al., 2014). Depending on the site of infection, different clinical forms of toxoplasmosis, such as neuro-toxoplasmosis and neurological disorders (McConkey et al., 2013), ocular toxoplasmosis (Ferreira et al., 2014), and congenital toxoplasmosis, arise (Chung et al., 2018). Some other clinical manifestations of this disease have been reported but seem uncommon (Saxena et al., 2018). These clinical entities place *T. gondii* and toxoplasmosis in a prominent position in contemporary medicine (Furtado et al., 2011; Subauste et al., 2011).

The global serological prevalence of infection by *T. gondii* is high, affecting about one-third of the world’s population (Pappas et al., 2009; Foroutan-Rad et al., 2016). However, the rates vary according to the region and the habits of the population. South American countries such as Brazil present infection rates above 60% (Dubey et al., 2012), while some Asian countries such as Taiwan have less than 10% (Chiang et al., 2012). Socioeconomic conditions such as illiteracy, low family income, and unemployment influence high infection rates (Meier et al., 2016; Mareze et al., 2019).

Different routes contribute to the transmission of *T. gondii* to humans. Although congenital transmission is an essential route of dissemination to fetuses and newborns, the infection acquired after birth can be due to different factors, including the consumption of raw or undercooked meat, the ingestion of contaminated vegetables and water, as well as contact with soil and cat feces (Furtado et al., 2011; Dubey et al., 2012; Ferreira et al., 2014). Transfusion of blood components and organ and tissue transplantation is also among the potential transmission routes (Foroutan-Rad et al., 2016; Dard et al., 2018).

Laboratory diagnosis of toxoplasmosis is essentially serological, but a set of molecular methods detect *T. gondii* genomic DNA in different biological samples (Liu et al., 2015; Dard et al., 2016). Serology detecting IgM and IgG anti-*T. gondii* antibodies and PCR (conventional PCR, Nested PCR, Real-Time PCR) are standard methods in determining acute and chronic infection (Okay et al., 2009; Saki et al., 2019; Murata et al., 2020).

*T. gondii* infection and the different clinical forms of toxoplasmosis have gained importance as public health issues. The risks that this parasite present to humans is considerable, especially to HIV-AIDS patients (Laboudi, 2017), cancer patients (Anvari et al., 2019), organ recipients (Robert-Gangneux et al., 2018), pregnant women, fetuses, and neonates (Chung et al., 2018). Therefore, understanding the different aspects of *T. gondii* infection contributes to better understanding the epidemiology of toxoplasmosis.

The susceptibility to *T. gondii* infection is heterogeneous and depends on parasite strains, environmental factors, and genetic traits of the human hosts. A set of human host genetic factors related to the acquisition risk of infection by *T. gondii* in diseased and healthy individuals is available in the literature. Polymorphisms of cell receptors such as Toll-Like Receptors (TLR-2: 2258G>A; TLR-4: 896A>G and 1196C>T, TLR-9: 2848G>A) (Wujcicka et al., 2017) and coreceptors (CCR5: 59029 AA or AG genotypes) (De Faria Junior et al., 2018) were associated with increased susceptibility. Genes and alleles controlling the immune response such as MICA and their HLA ligands (MICA-HLA: MICA*002~HLA-B*35), and KIR (KIR: KIR3DS1-Bw4-80Ile; KIR2DS1^+^/C2^++^ KIR3DS1^+^/Bw4-80Ile^+^) increase the risk of infection (Ayo et al., 2015; Ayo et al., 2016). High levels of the cytokines IL-6, IL-10, IL-12 and TNF-α were associated with increased susceptibility to infection and development of different clinical forms of toxoplasmosis (Maia et al., 2017).

A set of host risk factors are known (Pinto-Ferreira et al., 2019), but others need to be investigated further. This text discusses the potential contribution of histo-blood group carbohydrates from ABO, Lewis, and Secretor systems in infection by *T. gondii*. The carbohydrate profiles of these genetic systems have high diversity in the gastrointestinal tract (Henry, 2001), a site used by *T. gondii* as a route to infect humans (Dubey, 2009). These two independent events occur in the same organ, and it is reasonable to suppose that there is some biological relationship between them.

### Human ABO, Lewis and Secretor Histo-Blood Group Carbohydrates

The name human histo-blood systems bring together genetic systems whose genes control the expression of specific glycosyltransferases responsible for the biosynthesis of carbohydrate antigens in cells of the hematopoietic and non-hematopoietic tissues and exocrine secretions. The ABO, Lewis, and Secretor systems express part of the repertoire of carbohydrate antigens in the gastrointestinal tract (Henry, 2001; Nydegger et al., 2005). The glycosyltransferases encoded by FUT2 (Secretor), FUT3 (Lewis), and ABO (GTA, GTB) interact in the biosynthesis of ABO and Lewis carbohydrate antigens by adding specific monosaccharides to precursor oligosaccharides, creating new carbohydrate antigens. Specific polyclonal and monoclonal antibodies and some lectins recognize these carbohydrate antigens allowing the serological characterization of ABO and Lewis phenotypes in red blood cells, tissue phenotypes, and salivary Secretor and Nonsecretor phenotypes (Schenkel-Brunner, 2000; Imberty et al., 2003). The transfusion procedures and tissue and organ transplantation require the correct identification of the four main ABO phenotypes to match recipients and blood donors and recipients and organ donors (Edinur et al., 2015).

The frequencies of ABO, Lewis, and Secretor phenotypes are well established in all human populations, but variations in the rates occur worldwide (Daniels, 2013). The translational applications of these histo-blood group systems are under investigation, offering opportunities to develop new technologies, personalized medicine, and improve public health (Korchagina and Henry, 2015; Dotz and Wuhrer, 2016; Ryzhov et al., 2016). For example, the influence of the histo-blood group carbohydrates in the susceptibility to infection and immunity (Cooling, 2015; Brandão de Mattos and de Mattos, 2017; Stowell and Stowell, 2019a), in thrombosis, cardiovascular diseases, and metabolism (Stowell and Stowell, 2019b) as well as developing enteric virus vaccines (Ramani and Giri, 2019), are of importance in public health.

The histo-blood group carbohydrate antigens synthesized by ABO, Lewis, and Secretor glycosyltransferases are involved in the glycosylation of proteins and lipids in eukaryotic cells (Corfield, 2017; Brandão de Mattos et al., 2019). Six types of monosaccharides constitute the structure of histo-blood carbohydrates: β-D-Glucose (Glc), β-D-N-Acetylglucosamine (GlcNAc), β-D-Galactose (Gal), β-D-N-Acetylgalactosamine (GalNAc), α-Fucose (Fuc) and D-Mannose (Man). Some of them are the immunodominant sugars that define the antigenic epitopes recognized by polyclonal and monoclonal antibodies and lectins in serological phenotyping. GalNAc defines the A antigen, whereas Gal defines the B antigen in the ABO histo-blood group system. The Fuc defines the H, Le^a^, and Le^b^ antigens depending on their position in the precursor oligosaccharide chains (Gilliver and Henry, 2003).

Different precursor oligosaccharides serve as the substrate for the histo-blood group glycosyltransferase. The addition of the above monosaccharides to each precursor oligosaccharide by the ABO, Lewis, and Secretor glycosyltransferases gives rise to different carbohydrate antigens, which may differ in the spatial conformation and affinity to monoclonal antibodies (Imberty et al., 2003; De Mattos, 2016). Data from ABO, Lewis, and Secretor histo-blood group systems are present in **Table 1**. For more details of biochemical and structural characteristics of histo-blood group carbohydrates, readers should refer to Schenkel-Brunner (2000) and De Mattos (2016).

**Table 1:** ABO, Lewis, and Secretor histo-blood group systems data. Modified from De Mattos (2016).

Systems	HGNC^1^	ISBT^2^	Chromosomes	Glycosyltransferase (Abbreviations)	IUBMB^3^ Code	Immunodominant sugar	Antigens
Secretor	*FUT2*	SE	19q13.3	α1,2-Fucosyltransferase (FUTII)	EC 2.4.1.69	Fuc	H type 1
Lewis	*FUT3*	LE	19p13.3	α1,3/4-Fucosyltransferase (FUTIII)	EC 2.4.1.65	Fuc, GalNAc, Gal	Le^a^, Le^b^, ALe^b^, BLe^b^
ABO	*ABO*	ABO	9q.34.1	α1,3-N-Acetylgalactosaminyltransferase (GTA)	EC 2.4.1.40	GalNAc	A type 1, A type 2
				α1,3-N-Galactosyltransferase (GTB)	EC 2.4.1.37	Gal	B type 1, B type 2

^1^HGNC, Human Genome Nomenclature Committee; ^2^ISBT, International Society for Blood Transfusion; ^3^International Union of Biochemistry and Molecular Biology.

The biosynthesis of histo-blood group carbohydrates in the gastrointestinal tract and exocrine secretions is a complex biological process. Depending on the presence, absence or combination of ABO, Lewis, and Secretor glycosyltransferases, variable levels of histo-blood group carbohydrate will be synthesized at the gastrointestinal tract and in the exocrine secretions. For example, the FUTII glycosyltransferase synthesizes the H carbohydrate antigen, the primary substrate for GTA, GTB, and FUTIII glycosyltransferases synthesize new carbohydrate antigens. Since Secretors carry a functional FUTII glycosyltransferase synthesizing H carbohydrate antigen, they can express high variability of histo-blood group carbohydrates compared to Nonsecretor, which has no functional FUTII glycosyltransferase (Henry et al., 1995; Imberty et al., 2003). **Table 2** shows the relative quantities of histo-blood group carbohydrates expressed in the exocrine secretions and mucosal tissues according to the combination of the ABO, Lewis and Secretor functional glycosyltransferases, as proposed by Perry and colleagues (Perry et al., 2007).

**Table 2:** The relative level of histo-blood group carbohydrates expressed in tissues and exocrine secretions according to the combination of histo-blood group glycosyltransferases. Modified from Perry et al., 2007.

Systems	Glycosyltransferases	OP type 1	H	Le^a^	Le^b^	A type 1	B type 1	ALe^b^	BLe^b^	RBC phenotypes
ABO, Lewis, Secretor	FUTII + FUTIII + GTA	(+)	(+)	+	+++	+++	0	++++	0	A Le(a-b+)
ABO, Lewis, Secretor	FUTII + FUTIII + GTB	(+)	(+)	+	+++	+++	++	0	++++	B Le(a-b+)
ABO, Lewis, Secretor	FUTII + FUTIII + GTA + GTB	(+)	(+)	+	+++	+++	++	++++	++++	AB Le(a-b+)
ABO, Lewis, Secretor	FUTII + FUTIII	(+)	(+)	+	+++++					O Le(a-b+)
ABO, Lewis, Secretor	FUTII + GTA	+	++			+++++				A Le(a-b-)
ABO, Lewis, Secretor	FUTII + GTB	+	++				+++++			B Le(a-b-)
ABO, Lewis, Secretor	FUTII + GTA + GTB	+	++			+++++	+++++			AB Le(a-b-)
ABO, Lewis, Secretor	FUTII	+	+++++							O Le(a-b-)
ABO, Lewis, Non-Secretor	FUTIII + GTA	+		+++++						A Le(a+b-)
ABO, Lewis, Non-Secretor	FUTIII + GTB	+		+++++						B Le(a+b-)
ABO, Lewis, Non-Secretor	FUTIII + GTA + GTB	+		+++++						AB Le(a+b-)
ABO, Lewis, Non-Secretor	FUTIII	+		+++++						O Le(a+b-)

OP, Oligosaccharide precursor; RBC, Red blood cell; (+): Very low expression; +, ++: Low expression; +++, ++++ Moderate expression; +++++: High expression.

ABO and Lewis red blood cell phenotyping predict part of the carbohydrate profile expressed in the gastrointestinal tract. However, a combination of serological, histological analysis such as immunohistochemistry and molecular methods are required to determine the correct tissue phenotypes and the level of expression of histo-blood group carbohydrates (Henry et al., 1994; Henry, 2001). Determining the correct histo-blood group carbohydrate profile is crucial to establish correct associations with infections and diseases (Henry et al., 1995). These approaches were critical in studies investigating the human gastrointestinal pathogen *Helicobacter pylori*, which is able to bind the histo-blood group carbohydrate Lewis b (Le^b^) expressed in the stomach mucosae (Borén et al., 1993; Martins et al., 2006).

### Associations Between *T. gondii* Infection and ABO, Lewis, and Secretor Histo-Blood Groups

The histo-blood group carbohydrates and *T. gondii* infection studies mostly focused on of associations enrolling seropositive patients and seronegative controls. Some reported statistically significant differences favoring one or more ABO phenotypes, while others did not find these differences. The majority of the studies examined only the ABO system and neglected the Secretor and Lewis histo-blood groups.

Positive and negative associations have been reported in the literature in the last three decades. A study carried out in Norway enrolling volunteers from different areas found a high proportion of anti-*T. gondii* antibodies in B (28.8%) and AB (18.0%) phenotypes (Midtvedt and Vaage, 1989). These frequencies were higher than those from the general population (9.1% and 3.4%, respectively). These authors proposed that the B histo-blood group carbohydrate, which contains two galactose units in its terminal structure, might be an important receptor for *T. gondii* in the gastrointestinal tract.

Subsequent reports were published confirming the observations from the study from Norway. In one of them, the authors found IgG anti-*T. gondii* antibodies in 75% of Cuban blood donors from AB histo-blood phenotype, a rate elevated compared to the general population (3.6%) (Lópes et al., 1993). Another study from Russia reported that 54% of the blood donors having anti-*T. gondii* antibodies were from the AB histo-blood group phenotype, and 27% were from the O histo-blood group phenotype (Zhiburt et al., 1997). Two other studies in the Czech Republic enrolling military personnel and in the Philippines enrolling individuals from urban and suburban areas also found an association between IgG anti-*T. gondii* antibodies with B and AB histo-blood group phenotypes (Kolbekova et al., 2007; Salibay et al., 2008). The data from these studies align with experimental observations showing galactose as an important monosaccharide in the interactions between humans and *T. gondii*. Galactose is a common component of many oligosaccharides involved in the glycosylation of proteins and lipids, but its presence is not exclusive to the B histo-blood group carbohydrate structure (Coelho et al., 2015).

Reports of no association between infection by *T. gondii* and ABO histo-blood group system are found in the literature. In Tanzania, Africa, statistically significant differences were not found in ABO phenotypes frequencies and anti-*T. gondii* antibodies in blood donors (Gill, 1985). Another study enrolling 4,000 pregnant French women found no differences in the frequencies of the ABO phenotypes in those seropositive and seronegative (Lécolier et al., 1990).

Our group carried out studies in northwestern São Paulo State, Brazil, a region with high infection rates by *T. gondii* (Gonçalves et al., 2010; Ferreira et al., 2014). In one of them, investigating only the ABO histo-blood group phenotypes, no statistically significant differences were observed in the proportions of seropositive and seronegative pregnant women (Rodrigues et al., 2011). No associations were observed in a second study, combining the ABO and Secretor histo-blood group phenotypes (Brandão de Mattos et al., 2008). This study considered that the expression of ABO histo-blood group carbohydrates in the gastrointestinal tract is under control of the FUT2 gene. In another one, we investigated the profile of histo-blood group carbohydrates resulting from the integrated action of the glycosyltransferases encoded by *FUT2*, *FUT3*, and *ABO* genes (Nakashima et al., 2019). This study showed that the Leb carbohydrate confers some protection against infection by *T. gondii*. We recently investigated only the ABO histo-blood group phenotypes in a series of 1,730 blood donors, and no evidence of an association between seropositivity and the ABO phenotypes was found (Unpublished). In the light of these observations, the biological role of ABO, Lewis, and Secretor histo-blood group carbohydrates in the infection by *T. gondii* remains unsolved and requires new strategies for investigations. **Table 3** shows the data of positive and negative associations between ABO, Lewis, and Secretor histo-blood group carbohydrates and infection by *T. gondii*.

**Table 3:** Studies of the association between ABO, H, Lewis, and Secretor histo-blood group carbohydrates and infection by *Toxoplasma gondii* and toxoplasmosis.

Place/Positive	Source	N	Seropositivity		Methods	HBG systems	Association	Phenotypes	Effects	References
			n	%						
Tanzania	Blood donors	208	79	38.0	IHA*	ABO	No	—	—	Gill, 1985
Norway	Volunteers	1,788	395	22.1	TcT^€^	ABO	Yes	B and AB	Susceptibility	Midtvedt & Vaage, 1989
France	Pregnant (MR)	4,000	3,127	78.0	NR^#^	ABO	No	—	—	Lécolier et al., 1990
Russia	Patients	38	15	39.5	NR^#^	ABO	Yes	A	Susceptibility	Teplinskaia & Kaliberdina, 1992
Cuba	Blood donors	1,036	684	66.0	IgG ELISA	ABO	Yes	AB	Susceptibility	López et al., 1993
Russia	Blood donors	323	NR	NR	NR	ABO	Yes	B and AB	Susceptibility	Zhiburt et al., 1997
Czech Republic	Military	3,250	748	23.0	IgM, IgG ELISA	ABO	Yes	B and AB	Susceptibility	Kolbekova et al., 2007
					CFT^&^					
Philippines	Urban, suburban residents	140	38	27.1	LAT^¥^	ABO	Yes	B	Susceptibility	Salibay et al., 2008
Brazil	Pregnant women	367	182	49.6	IHA*	ABO, Secretor	No	—	—	Brandão de Mattos et al., 2008
Egypt	Blood donors	260	155	59.6	IgG ELISA	ABO	Yes	O	Susceptibility	Elsheikha et al., 2009
Brazil	Pregnant women (MR)	1006	645	64.1	IgM, IgG ELISA	ABO	No	—	—	Rodrigues et al., 2011
Taiwan	Blood donors	1,783	166	9.3	IgM, IgG ELISA	ABO	No	—	—	Chiang et al., 2012
Czech Republic	Military	491	154	31.4	IgM, IgG ELISA	ABO	No	—	—	Flegr et al., 2013
Iraq	Blood donors	400	131	32.7	IgM, IgG ELISA	ABO	Yes	AB	Susceptibility	Mahmood et al., 2013
			136	34.0	LAT					
Iran	Blood donors	1,480	286	19.3	IgM, IgG ELISA	ABO	No	—	—	Sarkari et al., 2014
Iran	Blood products	250	59	23.6	IgM, IgG ELISA	ABO	Yes	B, AB	Susceptibility	Shaddel et al., 2014
Iran	Blood donors	375	94	25.0	IgM, IgG ELISA	ABO	No	—	—	Modrek et al., 2014
Iran	Blood donors	500	160	32.0	IgM, IgG ELISA	ABO	Yes	B	Susceptibility	Mahmoudvand et al., 2015
Côte d’Ivoire	Blood donors	106	68	64.1	IgM, IgG ELISA	ABO	No	—	—	Siransy et al., 2016
Iran	Blood donors	491	200	40.7	IgM, IgG ELISA	ABO	No	—	—	Sadooghian et al., 2017
Iran	Blood donors	285	48	16.8	IgM, IgG ELISA	ABO	No	—	—	Moshfe et al., 2018
Iraq	Miscarriage	200	67	33.5	IgM LAT, ELISA	ABO	No	—	—	Smael et al., 2018
Egypt	Blood donors	276	150	54.3	IgM, IgG ELISA	ABO	No	—	—	Abd El Wahab et al., 2018
Brazil	Pregnant women	244	158	64.7	IgM, IgG ELISA	ABO, Lewis, Secretor	Yes	Le(a-b+)	Resistance	Nakashima et al., 2019
Brazil	Blood donors	1,730	835	48.3	IgM, IgG ELISA	ABO	No	—	—	Nakashima et al. (Unpublished data)

*IHA, Indirect Hemagglutination; ^€^TcT, Toxoplasmin Cutaneous Test; ^&^CFT, Complement Fixation Test; ^¥^LAT, Latex Agglutination Test; ^#^Non Referred; MD, Data from Medical Records.

### Experimental Demonstrations That *Toxoplasma gondii* Binds Carbohydrates

The investigation of potential biological relationships between the infection by *T. gondii* and the ABO, H, Lewis, and Secretor histo-blood group carbohydrates is attractive. A set of experiments carried out *in vitro* demonstrated that these histo-blood group carbohydrates act as receptors favoring the attachment of microorganisms at the gastrointestinal mucosae such as *Helicobacter pylori* (Borén et al., 1993), *Candida albicans* (Cameron and Douglas, 1996), Rotavirus (Sun et al., 2018), and Norovirus (Esseili et al., 2019). These microorganisms bind histo-blood group carbohydrates in a specific manner, increasing the susceptibility of individuals who express some of these oligosaccharide structures. However, *in vivo* experiments demonstrating the attachment of *T. gondii* to ABO, H, Lewis, and Secretor histo-blood group carbohydrates are scarce.

Some studies demonstrated that carbohydrates are involved in one or more steps of the cell invasion process exploited by *T. gondii*. One of them investigated the ultrastructural localization of carbohydrate residues and sugar-binding sites in the rhoptries from *T. gondii* tachyzoites (De Carvalho et al., 1991). Their results suggested that these sugar-binding sites play an essential role during the process of *T. gondii*-host cell interaction. Another one demonstrated that *T. gondii* micronemal protein MIC1, one of the first proteins released in the cell invasion process, is a critical lactose-binding lectin (Lourenço et al., 2001). *T. gondii* induces conformational changes in the microneme complex TgMIC4. This complex can bind various galactose-containing carbohydrates (Marchant et al., 2012; Santos et al., 2015). Chinese hamster ovary cells deficient in sialic acid, a widely distributed monosaccharide on the cell surface of all vertebrates, are resistant to infection by *T. gondii* (Monteiro et al., 1998). These authors proposed that sialic acid is a critical monosaccharide for cell attachment and invasion of *T. gondii*. A recent paper supports their data that *T. gondii* uses the sialic acid-binding protein-1 localized on its outer membrane to bind sialic acid for host cell attachment and invasion (Xing et al., 2020 **)**. These reports reinforce the potential role of the host’s oligosaccharides in the host-*T. gondii* interactions.

Oligosaccharide chains containing galactose in the inner structure and the terminal monosaccharide are the precursors for ABO, Lewis, and Secretor histo-blood group carbohydrates expressed in the gastrointestinal tract (De Mattos, 2016). The combined action of the glycosyltransferases encoded by the *ABO*, *FUT1*, *FUT2*, and *FUT3* genes in the gastrointestinal tract create a carbohydrate repertoire that acts as receptors containing galactose that modify the susceptibility or resistance to pathogens **(** Henry, 2001). It is reasonable to speculate the potential contribution of the repertoire of histo-blood group carbohydrates as a host’s risk factors for infection by *T. gondii*. However, experiments demonstrating that *T. gondii* tachyzoites or oocysts bind specific histo-blood group carbohydrates are required.

### The Significance of Positive and Negative Associations Between ABO, Lewis and Secretor Histo-Blood Group Systems and *T. gondii* Infection

In the general context, studying associations between biomarkers and infectious diseases is a good starting point to understand the epidemiology and pathophysiology of a disease. Carrying out experimental demonstrations resolving the contribution of a genetic trait in the susceptibility or resistance to disease reinforces the importance of association studies, providing a background concerning the contributions of biomarkers in disease screening, diagnosis, prognosis, and therapy (Timpson et al., 2018). The reported associations between ABO, Lewis, and Secretor histo-blood group systems and susceptibility to infection by pathogenic microorganisms are crucial to initiate experimental investigations aiming to demonstrate the contribution of the histo-blood group carbohydrates as receptors for pathogens (Cooling, 2015; Stowell and Stowell, 2019a).

In this scenario, experimental demonstrations that histo-blood group carbohydrates can act as receptors or coreceptors for *T. gondii* in the gastrointestinal tract would allow translational applications in areas with a high prevalence of this parasite. Maybe these carbohydrates could be helpful to estimate the risk for the different clinical forms, disease progression, and personalized treatment, and prevention of toxoplasmosis, especially in contemporary personalized medicine.

Without experimental demonstrations, the associations between the ABO, Lewis, and Secretor histo-blood group systems and infection by *T. gondii* weaken. The studies investigating only the ABO red blood cell phenotypes require at least two considerations. Firstly, *T. gondii* does not use erythrocytes as a host cell but infects nucleated cells. Secondly, the ABO tissue phenotypes result from the action of FUTI, FUTII, FUTIII, GTA and GTB glycosyltransferases (Henry and Samuelsson, 2000). The presence, absence, or combination of these glycosyltransferases affects the tissue carbohydrate profiles, varying according to ethnicity (Henry, 2001). Genetic events such as single nucleotide polymorphisms, deletions, insertions, and recombinations might affect the efficiency of the encoded glycosyltransferases, adding complexities to the carbohydrate profiles (De Mattos, 2016 **)**. This set of events can misinterpret the associations between the infection by *T. gondii* and ABO, Lewis and Secretor histo-blood group systems.

The contradictory data that emerged from the studies enlisted in **Table 3** might be due to different reasons. Individuals such as pregnant women, volunteers, military personnel, urban and suburban area residents, and blood donors were enrolled. The number of individuals in each study varies from a few tens to thousands, and the inclusion and exclusion criteria are not clear. Furthermore, the detection of anti-*T. gondii* antibodies did not follow standard laboratory tests. This heterogeneity creates contradictory results and weakens the comparisons among them.

From 25 studies in **Table 3**, eighteen used the ELISA method to identify IgM and IgG anti-*T. gondii* antibodies. ELISA is an excellent method to detect antigens and antibodies in infectious diseases due to its excellent sensitivity and specificity. Besides being cheaper, it indicates the serological immune status of the infected and noninfected individual at low costs (Shah and Maghsoudlou, 2016). However, limitations of serological tests such as ELISA to detect anti-*T. gondii* antibodies may also be related to different antigens (native versus recombinant) present in the commercial kits (Zhang et al., 2017). Some studies have shown that some commercial kits failed to detect low levels of IgG anti-*T. gondii* (Douet et al., 2019).

Considering that *T. gondii* has high genetic variability from one region to another, recombinant antigens present in commercial kits can vary among manufacturers affecting their sensitivity and specificity. Commercial kits manufactured with different recombinant antigens may fail to detect some *T. gondii* strains, especially in South American countries, due to the diversity of strains (Arranz-Solís et al., 2019). These differences might produce false-positive or false-negative results and fail to identify individuals with a reagent and non-reagent serology (Wilson et al., 1997). These characteristics might contribute to the contradictory data reported by studies associating histo-blood group carbohydrates and infection by *T. gondii*.

The amount of histo-blood group carbohydrates linked to proteins and lipids varies in exocrine secretions and the blood plasma depending on epistatic interactions between histo-blood group FUTI, FUTII, FUTIII, GTA and GTB glycosyltransferases (Henry et al., 1997; Achermann et al., 2005; Cooling, 2015). A previous study reported quantities of ALe^b^ carbohydrate three times higher than A type 1, representing almost 3% of the neutral glycosphingolipids in the blood plasma (Lindstrom et al., 1992). One can speculate that these quantitative differences impact the susceptibility or resistance to infections by pathogens such as *T. gondii*.

Old reports showed that some human pathogens such as the juvenile forms of *Schistosoma mansoni* (Goldring et al., 1976), *Fasciola hepática* (Ben-Ismail et al., 1982) and *Toxocara canis* (Smith et al., 1983) could synthesize histo-blood group carbohydrates or adsorb them to their surface from human blood and exocrine secretions. These authors propose that adsorbing histo-blood group carbohydrates could represent a strategy of evasion from innate and adaptive immune responses giving parasites an advantage for survival in the host. Without experimental evidence showing that *T. gondii* explores these strategies in humans, the proposition made by the Norwegian group that B histo-blood group carbohydrate facilitates the infection by this parasite remains speculative.

An alternative explanation to the associations shown in **Table 3** could be related to the presence of α-Gal epitopes in *T. gondii* tachyzoites and oocysts. The α-Gal epitope (Galα1→3Galβ1→4GlcNAc-R) is an oligosaccharide synthesized by the α-1,3-Galactoyltransferase whose gene α1,3GT, was lost by humans and anthropoid primates during evolution (Galili, 1997). The α-Gal epitope is structurally related to B histo-blood group carbohydrate (Galα1→3[Fucα1→2]Galβ1→4GlcNAc-R) (Cabezas-Cruz et al., 2017). The absence of this epitope in humans coincides with their ability to produce a natural anti-α-Gal antibody such as IgG, IgM and IgA isotypes, possibly due to the stimulus given by the α-Gal epitope present in gut microorganisms (Huai et al., 2016). The production of an anti-α-Gal antibody by humans is independent of ABO, Lewis and Secretor histo-blood group systems. However, its plasma level is low in individuals from B blood group, making them more prone to infectious diseases such as malaria and tuberculosis (Cabezas-Cruz et al., 2017). This observation does not align with the infection by *T. gondii* due to the lack of α-Gal epitope in tachyzoites (Hodžić et al., 2020). Considering that the infection by *T. gondii* after birth occurs essentially by the gastrointestinal tract, studies aiming to demonstrate α-Gal epitope in this parasite would help clarify the associations with B and AB blood groups.

## Exploring Animal Models

Experimental animal models help understand many aspects of the pathophysiology of infectious and parasitic diseases. Although many experimental studies focus on the strategies used by *T. gondii* to invade nucleated cells (Subauste, 2012; Dunay et al., 2018), this approach exploring relationships between this parasite and histo-blood group carbohydrates are scant. These models could help understand how *T. gondii* explores histo-blood group carbohydrates as potential receptors or coreceptors and favors new strategies for managing infection, disease treatment, and prevention.

Using Rhesus monkeys as an animal model, it was possible to understand some aspects of *H. pylori* and histo-blood group carbohydrates (Lindén et al., 2008). This human pathogen uses the gastrointestinal tract as a route of infection. Primates such as Rhesus monkeys express ABO, Lewis and Secretor histo-blood group carbohydrates in the gastrointestinal tract and exocrine secretions besides red blood cells (Blancher and Socha, 1997). Therefore, they could be helpful as an animal model to experimentally verify if any particular histo-blood group carbohydrate facilitates the infection by *T. gondii* through the gastrointestinal tract. These studies could also evaluate if the predisposition to infection by *T. gondii* linked to a particular histo-blood group carbohydrate depends on age, gender, ways of transmission, and virulent and nonvirulent strains. Data arising from these animal models could offer insights for translational applications in prevention, diagnosis, and outcome of disease and treatment.

## Concluding Remarks

Studies comparing the ABO, Lewis, and Secretor histo-blood group systems and infection by *T. gondii* reported contradictory results. Experimental demonstrations that galactose is involved in the cell invasion by this parasite could, per se, explain these associations. However, this monosaccharide is widespread in many human cells, tissues, and exocrine secretions as part of the glycosylation process, and it is not present only in the B histo-blood group carbohydrate. Galactose is a unit of sugar composing many of the precursor oligosaccharide substrates for the histo-blood group glycosyltransferases. Therefore, the biological role of histo-blood group carbohydrates as receptors or coreceptors to *T. gondii* remains unexplained and requires further investigations.

The histo-blood group glycosyltransferases have evolved to synthesize and diversify a portion of oligosaccharides expressed in the gastrointestinal tract. This strategy represents a crucial biological process for changing potential receptors for pathogenic microorganisms (Henry, 2001). The FUTII glycosyltransferase diversifies the carbohydrate structures and reduces the extension of histo-blood group carbohydrate chains in secretors compared to nonsecretors (Angstrom et al., 2004). Units of galactose are present in the terminal structure and inner core of histo-blood group carbohydrate antigens. Better understanding these characteristics of histo-blood group carbohydrates may improve understanding of how ABO, Lewis, and Secretor contribute to infection by *T. gondii*.

Future studies focusing on the binomial histo-blood group carbohydrates and *T. gondii* infection should address questions regarding the role of these carbohydrates in the infection and the different clinical forms of toxoplasmosis. Also, experimental tests must determine if *T. gondii* virulent and nonvirulent strains bind histo-blood carbohydrates in a specific manner. Answering these questions could offer some translational applications for susceptibility, treatment, and prevention. The investigation of these factors offers an attractive field for research to understand the contribution of histo-blood group carbohydrates in the infection by *T. gondii*.

## Data Availability Statement

The raw data supporting the conclusions of this article will be made available by the authors, without undue reservation.

## Author Contributions

LM and CB design the study and wrote the manuscript. AF, KY, and FN collected the data from the literature and drew the tables in the manuscript. All authors contributed to the article and approved the submitted version.

## Funding

This work was supported by FAPESP São Paulo Research Foundation (#2009/17540-2; 2012/07716-9 to LM; 2012/07750-2 to FN), by CAPES (to AF) and by CNPq (to CB).

## Disclaimer

The opinions, assumptions, and conclusions or recommendations expressed in this material are the authors’ responsibility and do not necessarily reflect the views of the FAPESP. The funders had no role in study design, data collection and analysis, decision to publish, or manuscript preparation.

## Conflict of Interest

The authors declare that the research was conducted in the absence of any commercial or financial relationships that could be construed as a potential conflict of interest.
